# Predictors of positive blood cultures in critically ill patients: a retrospective evaluation

**DOI:** 10.3325/cmj.2012.53.30

**Published:** 2012-02

**Authors:** Marco Previsdomini, Massimiliano Gini, Bernard Cerutti, Marisa Dolina, Andreas Perren

**Affiliations:** 1 Intensive Care Unit, Ospedale Regionale Bellinzona e Valli, Bellinzona, Switzerland; 2 Department of Internal Medicine, Ospedale Regionale Bellinzona e Valli, Bellinzona, Switzerland; 3 Unit of Development and Research in Medical Education, Faculty of Medicine, University of Geneva, Geneva, Switzerland; 4 Cantonal Institute of Microbiology, Bellinzona, Switzerland; The first two authors contributed equally.

## Abstract

**Aim:**

To identify predictors of bacteremia in critically ill patients, to evaluate the impact of blood cultures on the outcome, and to define conditions for breakthrough bacteremia despite concurrent antibiotic treatment.

**Methods:**

A descriptive retrospective study was performed over a two-year period (2007-2008) in the medico-surgical Intensive Care Unit (ICU) of the San Giovanni Hospital in Bellinzona, Switzerland.

**Results:**

Forty-five out of 231 patients (19.5%) had positive blood cultures. Predictors of positive blood cultures were elevated procalcitonin levels (>2 µg/L, *P* < 0.001), higher severity scores (Simplified Acute Physiology Score II>43, *P* = 0.014; Sequential Organ Failure Assessment >4.0, *P* < 0.001), and liver failure (*P* = 0.028). Patients with bacteremia had longer hospital stays (31 vs 21 days, *P* = 0.058), but their mortality was not different from patients without bacteremia. Fever (*t* > 38.5°C) only showed a trend toward a higher rate of blood culture positivity (*P* = 0.053). The rate of positive blood cultures was not affected by concurrent antibiotic therapy.

**Conclusions:**

The prediction of positive blood culture results still remains a very difficult task. In our analysis, blood cultures were positive in 20% of ICU patients whose blood was cultured, and positive findings increased with elevated procalcitonin levels, liver failure, and higher severity scores. Blood cultures drawn >4 days after the start of antibiotic therapy and >5 days after surgery could detect pathogens responsible for a new infection complication.

Sepsis is a common and threatening occurrence in the intensive care unit (ICU), where up to 35% of patients develop such a condition at some point during their stay (1). The associated mortality is 27% but exceeds 50% in cases of septic shock (1,2).

Blood cultures represent an important diagnostic tool, though they detect bacteremia in only about 50% of patients who are clinically suspected of having sepsis (2), with an even lower rate of positivity when drawn in the presence of ongoing antibiotic therapy (3-6). The presence of a blood pathogen represents a negative prognostic factor (7), but the isolation of such pathogen is crucial for verifying the appropriateness of antibiotic therapy, which is known to reduce morbidity and mortality (8,9). Furthermore, cultures of specific sites of suspected infection do not reliably predict the findings of blood cultures (10). Conversely, false-positive results from bacterial contaminants may lead to unnecessary antibiotic therapy, longer hospital stays (11), and selection of resistant microorganisms (12,13).

Most physicians have a low threshold for ordering blood cultures, regardless of concurrent antibiotics, whenever a patient develops a new fever. The same usually occurs in the case of a clinical decline that is potentially caused by infection or of laboratory signs of a worsening inflammatory state, bearing in mind that correlations are lacking between degree of fever, leukocytosis, and bacteremia (14-17).

We conducted a retrospective study in our multidisciplinary ICU in Switzerland to investigate the rate of positive blood cultures drawn from our ICU patients in case of temperature over 38.5°C and/or clinical decline with a concomitant worsening inflammatory state. We aimed to determine the influence of concurrent antibiotics, to identify predictors of bacteremia and conditions for breakthrough bacteremia despite antibiotics, and to compare our findings with those published mainly by US and Canadian university hospitals.

## Methods

### Setting

This descriptive retrospective study was performed in the ICU of the San Giovanni Hospital in Bellinzona, Switzerland. This is an eight-bed multidisciplinary teaching structure with about 750 adult admissions per year from internal medicine, oncology, general surgery (no cardiac surgery or organ transplantations), urology, orthopedic surgery, ear, nose, and throat, and gynecology. Considering the retrospective, non-interventional design of this quality assurance study, no informed consent was required by the Cantonal Ethics Committee.

### Study population, data collection and definitions

Patients aged ≥16 years who had at least one blood culture drawn in the ICU or within 24 hours before admission were retrospectively identified by our microbiology laboratory. Hospital charts were reviewed to collect personal data (age, sex), diagnosis at admission, the Simplified Acute Physiology Score (SAPS) II (at 24 hours from ICU admission) and Simplified Organ Failure Assessment (SOFA, computed on the day of sampling) (18,19), and lengths of stay in the ICU and in the hospital (also considering pre- and post-culture values). We recorded antibiotic status (dividing samples into pre-antibiotic and antibiotic blood culture groups, with the latter drawn with concurrent antibiotics), comorbidities (hepatic failure, active malignancy, diabetes mellitus), immunodeficiency (AIDS, immunosuppressive drugs, chemotherapy, steroids), and conditions on the day of the sampling potentially associated with the outcomes of the exams (body temperature, laboratory tests, invasive mechanical ventilation, indwelling venous/arterial or urinary catheters, surgical wounds). The number and timing of blood cultures, their results, interpretation, and data regarding antibiotic therapy administered before sampling were also obtained.

A blood culture was considered positive when it yielded *Staphylococcus aureus*, non-viridans group Streptococci (including group A or group B Streptococci), Enterococci, enteric gram-negative bacilli, *Pseudomonas* spp, *Bacteroides* spp, or fungi. Bacteria like *viridans* group Streptococci, coagulase-negative Staphylococci, *Propionibacterium* spp, *Corynebacterium* spp or *Bacillus* spp were considered pathogens (20) only if two separate blood cultures were positive and at least two SIRS criteria (21) were fulfilled. Otherwise, they were considered contaminants and blood cultures were not included in the bacteremic group.

### Specimen collection and processing

Blood samples were collected by nurses following a sterile procedure in accordance with the local protocol: 20 mL of blood was obtained for each blood culture, with 10 mL inoculated into each aerobic and anaerobic bottle. At least one blood culture (one bottle for aerobic growth and one for anaerobic growth) was drawn. Samples were processed by the Institute of Microbiology (Bellinzona) in a Bact/Alert 3D (bioMérieux SA, Marcy L’Etoile, France) automatic system at 35°C for at least seven days. Microbial growth was detected by continuously monitoring the CO_2_ production in the bottles. Identification of microorganisms and antibiotic susceptibility testing were performed according to standard laboratory operating procedures.

### Statistical analysis

Continuous values are shown as means ± standard deviations, and medians and ranges whenever the distribution was not normal. Categorical values were expressed as counts and percentages. To compare continuous values we used *t* test and Wilcoxon rank-sum test if the hypothesis of normality was rejected and for categorical values we used χ^2^ tests. We also considered analyses for every unit of observation (series of blood cultures) to neutralize discrepancies between patients with different number of examined septic episodes and different number of blood cultures per septic episode. A series of blood cultures was defined as all blood cultures obtained from one patient on the same day. This served to identify potential predictors of bacteremia. If at least one blood culture was positive (according to the previously mentioned criteria), the series was considered positive for bacteremia. The link between bacteremia and different factors was investigated with a logistic regression model, where age and presence of antibiotic therapy were systematically incorporated into the model to reduce the effect of potential biases. All analyses were performed with Spotfire S+^®^ 8.1 for Windows (TIBCO Software Inc. Palo Alto, CA, USA).

## Results

### Study population

**Figure 1:**
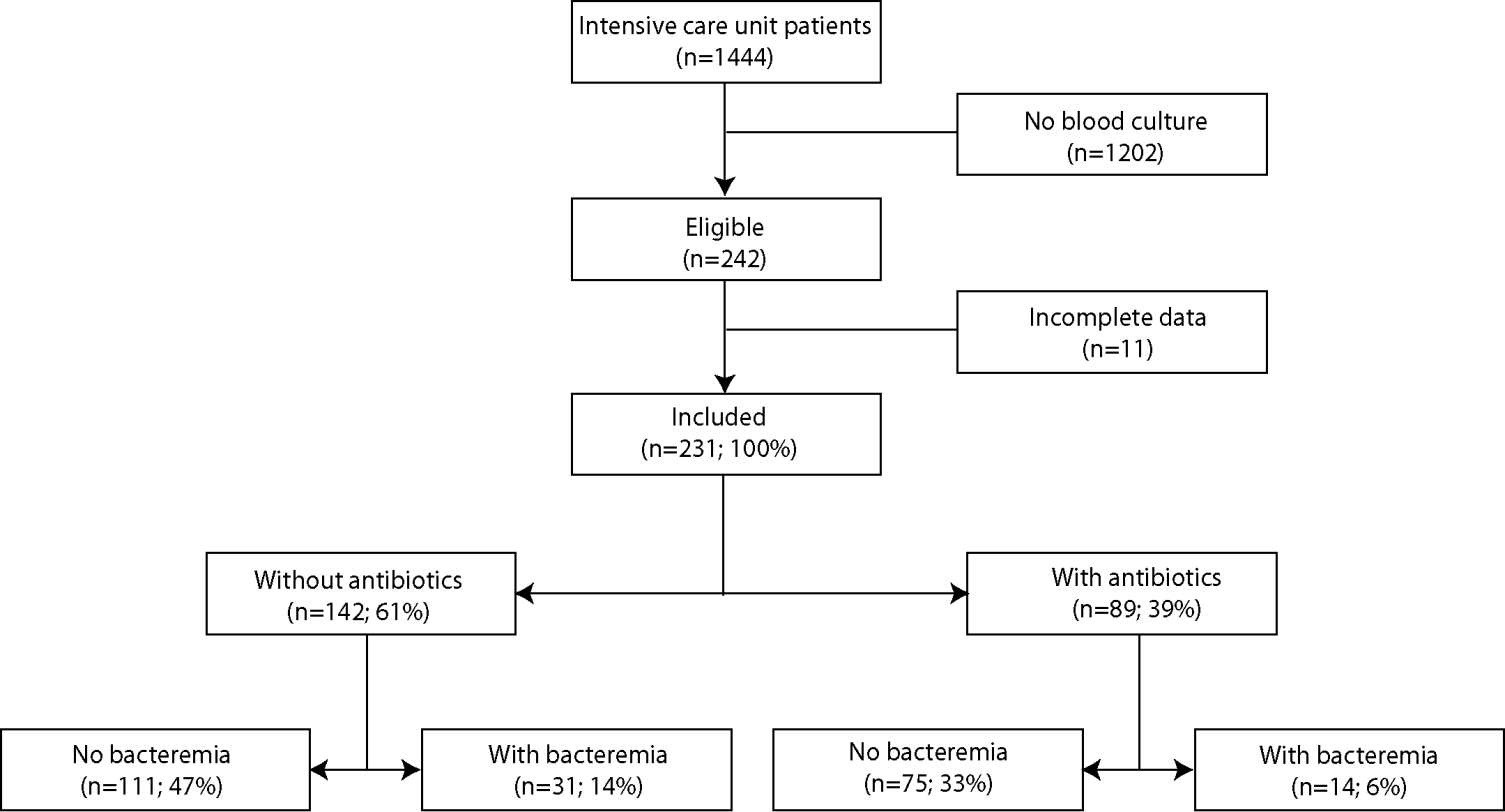
Study population and rates of negative and positive blood cultures

From 1444 patients admitted to the ICU from January 2007 to December 2008, 231 were eligible for the study (Figure 1), with 616 blood cultures and 310 series of blood cultures. There were 89 patients (39%) with 269 antibiotic blood cultures (44%) and 138 series of antibiotic blood cultures (45%). The blood of most patients (79%) was cultured on one day.

Negative blood cultures were found in 186 patients (81%), with 23 contaminations, 107 of whom were not given concurrent antibiotic therapy and 79 were. Forty-five patients had at least one positive blood culture (31 patients without and 14 patients with concurrent antibiotics), resulting in a true positive rate of 19.5% and an incidence of bacteremia of 3.1 per 100 ICU admissions.

The diagnosis at admission most frequently associated with positive results (69%) was sepsis/septic shock (Table 1; about 50% of patients with sepsis/septic shock had positive blood cultures). While the length of the ICU stay was unaffected by blood culture results, there was a trend toward longer hospital stays in bacteremic patients (Table 2). ICU and hospital mortality rates were 10% and 15%, respectively, and were not affected by positive blood culture results.

**Table 1:** Characteristics of patients with negative and positive blood cultures*

	No. (%) of patients			
	all (n = 231)	negative (n = 186)	positive (n = 45)	P
Age, mean ±SD (years)	65 ± 16	64 ± 16	67 ± 12	0.583
Sex:				
male	153 (66)	121 (65)	32 (71)	0.552
female	78 (34)	65 (35)	13 (29)	
SAPS II, mean ±SD	44.29 ± 19.78	42.44 ± 18.73	51.93 ± 21.75	0.007
Blood cultures performed, median (range)	2 (1-11)	2 (1-8)	2 (1-11)	<0.001
Diagnosis:				
sepsis/septic shock	63 (27)	32 (17)	31 (69)	<0.001
non-septic shock	10 (4)	9 (5)	1 (2)	0.439
cardiovascular disease	35 (15)	30 (16)	5 (11)	0.400
cardiac arrest	5 (2)	5 (3)	0 (0)	0.266
respiratory disease	45 (19)	42 (23)	3 (7)	0.016
gastrointestinal disease	6 (3)	6 (3)	0 (0)	0.222
neurological disease	18 (8)	15 (8)	3 (8)	0.754
trauma	8 (3)	8 (4)	0 (0)	0.157
surgery	25 (11)	23 (12)	2 (4)	0.205
psychiatric disease	3 (1)	3 (2)	0 (0)	0.391
others	13 (6)	13 (7)	0 (0)	0.070

*Abbreviations: SD – standard deviation; SAPS II – Simplified Acute Physiology Score II (18).

**Table 2:** Outcomes of patients with negative and positive blood cultures*

	Patients			
	all (n = 231)	negative (n = 186)	positive (n = 45)	*P*
Length of stay in days, median (range):				
intensive care unit	5 (1-125)	5 (1-125)	6 (2-46)	0.221
hospital	17 (2-194)	16 (2-136)	19 (2-194)	0.058
hospital before blood culture	1 (0-64)	1 (0-26)	0 (0-64)	0.445
hospital after blood culture	14 (1-192)	13 (1-135)	17 (1-192)	0.117
Mortality, n (%)				
intensive care unit	22 (10)	18 (10)	4 (9)	0.872
hospital	35 (15)	28 (15)	7 (16)	0.933

### Link between bacteremia and other factors

Positive blood cultures were associated with higher severity scores (SAPS II>43 at admission and SOFA score >4 the day of sampling; cut-off levels were set at the median of the observations) and hepatic failure (Table 3). A predictive factor were also procalcitonin levels above 2 µg/L, while fever showed only a trend toward a positive correlation. Fever higher than 38.5°C had a low positive predictive value (0.23; 95% confidence interval [CI], 0.14-0.33), while absence of fever had a higher predictive value (negative predictive value, 0.87; 95% CI, 0.81-0.91) without being discriminating. Actually, afebrile bacteremia (Tmax ≤38.5°C) was identified in 13% of units of observation.

**Table 3:** Potential predictors of bacteremia

Predictor Total	No. (%) of series of BCs (310)	No. (%) of series of BCs with bacteremia	Odds ratio (95% confidence interval)*	P*
Antibiotic therapy:				
yes	138 (45)	15 (11)	0.53 (0.27-1.04)	0.058
no	172 (55)	34 (20)	1	
Age (years):				
up to 55	87 (28)	8 (9)	1	0.063
56 to 65	64 (21)	17 (27)	3.33 (1.32-8.40)	
66 to 75	87 (28)	12 (14)	1.55 (0.60-1.55)	
≥76	72 (23)	12 (17)	1.77 (0.67-4.66)	
Sequential Organ Failure Assessment (19):				
>4	122 (39)	32 (26)	3.81 (1.95-7.44)	<0.001
≤4	188 (61)	17 (9)	1	
Simplified Acute Physiology Score II (18):				
>43	150 (48)	30 (20)	2.25 (1.16-4.38)	0.014
≤43	160 (52)	21 (12)	1	
Hepatic failure	32 (10)	10 (31)	2.81 (1.15-6.86)	0.028
Temperature (°C):				
>38.5	80 (26)	18 (22)	1.98 (1.00-3.94)	0.053
≤38.5	230 (74)	30 (13)	1	
White blood cells (G/L)				
>12	160 (52)	22 (14)	0.77 (0.40-1.45)	0.411
≤12	150 (48)	26 (17)	1	
C-reactive protein (mg/L):^†^				
>100	173 (57)	31 (18)	1.45 (0.76-2.80)	0.256
≤100	130 (43)	18 (14)	1	
Procalcitonin (μg/L):^‡^				
>2	53 (38)	15 (28)	9.68 (1.81-51.93)	<0.001
0.5-2	40 (28)	2 (5)	1	
<0.5	48 (34)	4 (8)	1.45 (0.23-9.35)	

*Adjusted by age and presence or absence of antibiotic therapy. BC – blood cultures.

†7 (2%) missing values.

‡169 (55%) missing values.

Factors that were not predictive of positive blood cultures were age (*P* = 0.063), major comorbidities (endocarditis [*P* = 0.379], pneumonia [*P* = 0.771], malignancy [*P* = 0.483], diabetes [*P* = 0.256]), immunodepression (chemotherapy [*P* = 0.599], immunosuppressive drugs [*P* = 0.139], steroids [*P* = 0.232]), and medical devices (central venous catheter [*P* = 0.508], urinary catheter [*P* = 0.139], drains [*P* = 0.829]). Indwelling arterial catheters and mechanical ventilation were actually correlated with negative blood cultures (odds ratio, 0.45; *P* = 0.020, and odds ratio, 0.46; *P* = 0.045, respectively). Seventy-three patients received invasive mechanical ventilation (36 of whom had major infections at admittance), with 242 blood cultures (176 antibiotic blood cultures, 73%). Seven of these patients (10% of patients and 9% of series of blood cultures) had positive blood cultures, independent of the length of mechanical ventilation (≤6 days vs >6 days, *P* = 0.101).

Positive blood cultures were not correlated with surgical wounds (*P* = 0.286). There were 36 surgical patients with 38 pre-antibiotic (31.7%) and 82 antibiotic blood cultures (68.3%). All 20 patients who were sampled within the first three postoperative days had negative blood cultures. All 4 patients with positive results had blood cultures drawn more than six days after surgery.

Bacteremia showed only a trend toward correlation with ongoing antibiotic therapy (*P* = 0.058), with no significant impact of its length: patients with negative blood cultures were treated for a mean of 6.2 days and patients with positive blood cultures were treated for a mean of 5.3 days. Nevertheless, 96.1% of blood cultures drawn within the first 72 hours were negative, and 83.3% of positive blood cultures were drawn more than 4 days after the start of antibiotic treatment. Bacteremic patients had a greater number of obtained blood cultures (Table 1) but also a greater number of series of blood cultures than non-bacteremic patients (1.7 for bacteremic patients and 1.2 for non-bacteremic patients, *P* = 0.009).

### Microbiology analysis

Pathogens were isolated from antibiotic blood cultures in 14 of 45 patients with positive blood cultures (Table 4). Gram-positive and Gram-negative bacteria were identified in the same proportions, and fungi were identified in three singular cases. *Staphylococcus aureus* was the only organism that grew in antibiotic blood cultures (two patients), despite normal immune status and adequate therapy (as assessed by in vitro testing) over four and five days, respectively. In four cases, while pre-antibiotic blood cultures remained sterile, we identified pathogens in antibiotic blood cultures: coagulase-negative Staphylococcus determining two catheter-related bloodstream infections (one from a peripheral venous line and one from a portacath); coagulase-negative Staphylococcus in a patient with superinfection of an aortic graft*;* and *Enterococcus faecium* and *Enterobacter aerogenes* in a patient with tertiary peritonitis. Two patients had different pathogens isolated from their pre-antibiotic and antibiotic blood cultures: *Enterococcus faecium* after a 7-day therapy for urosepsis (previous strain: *Escherichia coli*) and *Enterococcus faecium* and *Aerococcus viridans* after a 3-day therapy for acute cholangitis (previous strains: *Klebsiella oxytoca* and *Escherichia coli*).

**Table 4:** Pathogens isolated from blood

Organism	Patients with positive pre-antibiotic BCs*	Patients with positive antibiotic BCs*	Concurrent therapy	Duration of therapy (days)	Susceptibility to concurrent antibiotic therapy
**Gram-positive bacteria (n = 25):**					
*Staphylococcus aureus*	4	3	amoxicillin/clavulanate imipenem/cilastatin, vancomycin^‡^ amoxicillin/clavulanate, gentamycin	4 0*^¶^* 5,2	susceptible^†^ susceptible susceptible^†^
*Coagulase-negative Staphylococcus*	3	4	amoxicillin/clavulanate amoxicillin/clavulanate ceftriaxone ceftriaxone, metronidazole	9 3 2 6,2	resistant resistant resistant resistant
*Streptococcus pneumoniae*	3	0			
*Streptococcus bovis*	1	0			
*Enterococcus spp*	2	3	ceftriaxone, metronidazole^§^ amoxicillin/clavulanate imipenem/cilastatin^║^	3 7 6	resistant resistant resistant
*Aerococcus viridans*	0	1	ceftriaxone, metronidazole^§^	3	susceptible
*Gram-positive anaerobic cocci*	1	0			
**Gram-negative bacteria (n = 22):**					
*Escherichia coli*	12	1	amoxicillin/clavulanate, ciprofloxacin	0	susceptible
*Enterobacter spp*	1	1	imipenem/cilastatin^║^	6	susceptible
*Klebsiella spp*	3	1	amoxicillin/clavulanate	9	resistant
*Citrobacter spp*	2	0			
*Salmonella group E*	1	0			
**Fungi (n = 3):**					
*Candida glabrata*	0	2	tazobactam/piperacillin, vancomycin, fluconazole imipenem/cilastatin, fluconazole	11 11, 5	resistant resistant
*Candida pelliculosa*	0	1	imipenem/cilastatin, vancomycin^‡^	0	resistant
Total	33	17			

*Pre-antibiotic denotes blood cultures (BC) drawn without concurrent antibiotic therapy. Antibiotic denotes BCs drawn with concurrent antibiotic therapy. For microorganisms identified in the antibiotic BC type, duration and susceptibility to concurrent therapy are reported.

†In two cases, we observed persistent *Staphylococcus aureus* infection despite adequate antibiotic therapy.

‡*Staphylococcus aureus* and *Candida pelliculosa* were isolated in the same BC.

§*Enterococcus spp*. and *Aerococcus viridians* were isolated in the same BC.

║*Enterococcus spp*. and *Enterobacter spp*. were isolated in the same BC.

*¶*0 indicates that antibiotics were started on the same day but prior to sampling.

## Discussion

Our results showed that blood cultures obtained as part of an “extended” infectious work-up in a general ICU population had a limited chance of identifying pathogens. This confirms previous data reported in large US/Canadian tertiary hospitals (10,17,22,23). In our antibiotic-free population, 20% of the series of blood cultures was positive, and 11% was positive if obtained in the presence of a concurrent antibiotic therapy. Thus, only a trend toward difference was detected based on the antibiotic status.

The overall incidence of bacteremia was 3.1 per 100 ICU admissions, a rate comparable with that reported in an adult ICU in a 25-year observation period (24). Our results support the common notion that bacteremia in ICU patients is difficult to predict. We confirmed the results of other studies (3,25) that showed that fever alone cannot be considered a solid predictor of bacteremia, as it could also be an expression of non-infectious inflammatory reactions. Conversely, an absence of fever was associated with a low rate of blood culture positivity but was not discriminating, as 13% of series of blood cultures revealed microbiological growth.

C-reactive protein and elevated white blood cells had low predictive values for bacteremia, while elevated procalcitonin levels were correlated with positive blood culture findings, confirming its diagnostic value in general ICU settings (26) and in particular clinical scenarios (27,28). However, we also identified some cases of bacteremia with procalcitonin levels below 0.5 μg/L – a level only slightly above the cut-off value for ICU patients of 0.38 μg/L (26), which further strengthens the importance of a cautious clinical approach. The definite diagnosis of sepsis might not rely on a single measurement of procalcitonin but on a complete clinical and laboratory evaluation of the patient, with procalcitonin playing a considerable role.

Among the analyzed comorbid conditions, a predictor of positive blood culture results was liver failure. Acute and chronic liver diseases are known to be associated with an increased risk of bacteremia (29-31) and have been shown to be independent risk factors for the development of bacteremia in patients with community-acquired pneumonia (32). This feature has been correlated with impaired function of the hepatic reticuloendothelial system (33), and complement (34) and polymorphonuclear cells.

We identified no associations between bacteremia and immunodeficiency or diabetes, confirming the results of Grace et al (3). Interestingly, this is in contrast with the results reported by Stoeckle et al (35), who found a relative frequency of bloodstream infections to be more than 4 times higher in diabetic than in non-diabetic general inpatients.

Bacteremia was not associated with the presence of a surgical wound or mechanical ventilation. All blood cultures obtained within 72 hours after a surgical procedure remained sterile, which is in accordance with the finding that most early postoperative febrile episodes resolved spontaneously without confirmation of infection (36). Late blood cultures (6, 7, and 15 days after surgery) disclosed infective complications in only three cases. Blood cultures obtained from patients with mechanical ventilation had very low yields. This can be explained by patient selection (about 50% were ventilated for reasons other than infection) and the known low sensitivity of blood cultures for disclosing pathogenic microorganisms in ventilator-associated pneumonia (37).

We aimed to evaluate the utility of obtaining repeat blood cultures in patients receiving antibiotic therapy with new suspected septic episodes. Antibiotic blood cultures were very often negative, particularly if the pre-antibiotic blood culture had been negative; otherwise, the same pathogen was isolated as in the pre-antibiotic cultures. Nevertheless, in our analysis, which was not limited to the first three days of antibiotic therapy but included the entire ICU stay, we identified microorganisms not found in the pre-antibiotic samples in 6.5% of patients, demonstrating that this practice is not always useless and may even be very important for detecting either polymicrobial infections or new infection complications.

Bacteremic patients were investigated more often (ie, series of blood cultures) and with more blood cultures than non-bacteremic patients. However, the number of blood cultures drawn for each suspected septic episode was the same. Thus, our data do not challenge the current guidelines (20), which propose to limit the number of blood cultures drawn per suspected septic episode to two or three. A study in an ICU (38) demonstrated that limitation of blood cultures to up to three sets reduced the number of blood cultures ordered for suspected septic episodes from 3.0 to 2.2 with no untoward effects on patient care.

Patients with nosocomial bloodstream infections were shown in one study to have a worse outcome with a longer hospital stay and an attributable mortality rate (7). Other studies (23,39-41) did not find the association of bacteremia with excess mortality but did with longer hospital stays. Our data tend to confirm the latter, as we observed only a trend toward longer hospital stays, while the length of ICU stay and mortality rates were not different between the bacteremic and non-bacteremic patients. Eventually, we cannot exclude the possibility that our study population was too small for a meaningful assessment, as demonstrated by the inconsistence of mortality, illness severity, and prevalence of bacteremia. SOFA and SAPS II scores differed between bacteremic and non-bacteremic patients, but their discriminatory capacities should be checked prospectively (eg, by calculating receiver operating characteristic curves on a larger study population).

Our study has several limitations. The retrospective observational design implies selection biases and some missing values (eg, for procalcitonin). Nevertheless, we attempted to neutralize the effects of a different number of examined septic episodes and blood cultures per septic episode by opting for a unit of observation analysis (series of blood cultures) that considered all blood cultures drawn on a single day from one patient, which allowed us to adjust the impact of clinical characteristics of a given patient compared to another patient subjected to a different number of blood cultures. Also, data collection spanned over two years and the number of patients (ie, blood cultures) was modest, thus limiting the power of the study. Although blood cultures were collected following a local standard operating procedure, we cannot rule out some technical differences. This is a critical remark, as the yields of blood cultures are known to increase when appropriate measures are applied (eg, blood volume) (42). Triggers for blood culture collection were generally mentioned in clinical records, but we cannot exclude the possibility that there were more unlisted triggers. Similarly, we cannot verify how often blood cultures were not performed despite the presence of triggers for blood culture collection.

In conclusion, blood cultures in a general ICU represent an important diagnostic tool to identify bacteremia and to guide diagnostic and therapeutic choices. According to our analysis, their rate of positivity increases with illness severity (SAPS II and SOFA scores), elevated procalcitonin levels, and the presence of hepatic failure, but does not seem to be clearly influenced by the presence of ongoing antibiotic therapy. Early blood cultures drawn in the presence of ongoing antibiotics rarely identified pathogens not found in the pre-antibiotic blood cultures. Therefore, the decision to perform a repeat blood culture should rely on careful clinical judgment, bearing in mind that blood cultures drawn 4 days after an initially appropriate antibiotic therapy and 6 days after surgery can detect new pathogens, which mainly reveal new infection complications.
